# Supplementary material for: Endovascular baroreflex amplification and the effect on sympathetic nerve activity in patients with resistant hypertension: A proof-of-principle study
Source: PLoS One. 2021 Nov 16;16(11):e0259826. doi: 10.1371/journal.pone.0259826 (PMC8594823; doi:10.1371/journal.pone.0259826)
Supplement: S1 File — (PDF) [file pone.0259826.s002.pdf]

Inclusion criteria

Main study:

1. ≥18 years and ≤80 years of age.
2. Diagnosed with primary resistant hypertension.
3. Mean 24-h ambulatory systolic blood pressure (BP) ≥130 mmHg following at least 30 days on a stable antihypertensive medication regimen (no changes in medication or dose) and no more than 28 days prior to implantation.

Sub-study:

1. Eligible for the CALM-DIEM study and having passed all CALM-DIEM study in- and exclusion criteria at CALM-DIEM study screening.

Exclusion criteria

Main study:

1. Inability to provide written informed consent.
2. Known or clinically suspected baroreflex failure or autonomic neuropathy.
3. Known significant aortoiliac or common femoral artery disease that will prohibit safe femoral access.
4. Hypertension secondary to an identifiable and treatable cause other than sleep apnea (e.g. primary aldosteronism, renal artery stenosis, pheochromocytoma, Cushing's disease, coarctation of the aorta, hyper(para)thyroidism or an intracranial tumor).
5. Treatable cause of resistant hypertension including, but not limited to, improper BP measurement, volume overload, drug- or substance-induced hypertension (i.e.

NSAIDs, COX-2 inhibitors, oral contraceptives, steroids, cyclosporine, tacrolimus, erythropoietin, cocaine, amphetamines, sympathomimetics, licorice, chewing tobacco, ephedra, ma haung, bitter orange, and excessive alcohol intake) or other causes (i.e. non-adherence, inadequate drug doses, inappropriate drug combinations).

6. Arm circumference greater than 46 cm and/or BMI ≥45.
7. Chronic atrial fibrillation or recurrent atrial fibrillation with episode within the last 12 months.
8. History of bleeding complications with dual antiplatelet therapy in the past or known uncorrectable bleeding diathesis.
9. Current use of anticoagulation therapy, other than antiplatelet therapy. Examples include vitamin K antagonists and novel oral anticoagulants including apixaban, rivaroxaban, dabigatran and edoxaban.
10. Peptic ulcer disease with documented active ulcer or bleeding within the last year.
11. History of allergy to contrast media that cannot be managed medically.
12. Persistent symptomatic orthostatic hypotension (BP decrease >20/10 mmHg after standing).
13. Persistent symptomatic syncope documented to be related to hypertension within the last 6 months.
14. History of myocardial infarction or unstable angina within the past 3 months.
15. History of cerebrovascular disease within the past year, and National Institutes of Health Stroke Scale >5 or modified Rankin Scale >1.
16. Chronic kidney disease (eGFR calculated by the Modification of Diet in Renal Disease equation <45 ml/min).

17. Prior carotid surgery, radiation, or endovascular stent placement in either carotid region.
18. Severe valvular or structural heart disease (excluding left ventricular hypertrophy).
19. Severe chronic obstructive pulmonary disease (requiring 24-h oxygen or oral steroids), asthma, or severe pulmonary hypertension.
20. Uncontrolled diabetes mellitus with HbA1c ≥10%.
21. Active infection within the last month requiring antibiotics.
22. Uncontrolled co-morbid medical condition, including mental health issues, that would adversely affect participation in the trial.
23. Co-morbid condition that reduces life expectancy to less than 1 year.
24. Planned surgery or other procedure within the next 6 months requiring cessation of antiplatelet medications.
25. Pregnant or lactating females. For females of child-bearing potential, a positive pregnancy test within 7 days of the pre-randomization screening or refusal to use a medically accepted method of birth control for the duration of the trial.
26. Carotid duplex studies demonstrating significant obstructive carotid disease or significant plaque at the site of implantation or proximal to the carotid artery bulb or focal areas of atherosclerotic intimal thickness (IMT) of >1500 microns visualized in the region of the bifurcation (15 mm proximal and distal to the internal carotid atery (ICA) ostium).
27. Significant obstructive vascular disease, calcification or plaque of aortic arch and great vessels by ultrasound, CTA or MRA.
28. Renal artery stenosis >50% or systolic gradient >10 mmHg in borderline cases diagnosed by renal artery imaging in the last 36 months. Acceptable renal artery

imaging modalities include renal duplex, MRA, CTA, and selective or nonselective renal angiography depending on trial site diagnostic standards.

29. ICA lumen diameters <5 mm or >12.5 mm within the planned location of the implant placement via CTA or MRA. Evidence of landing zone restrictions, such as inadequate length, vessel tapering, and/or vessel curvature that would preclude safe placement of the implant.
30. Enrolled in a concurrent clinical trial for an investigational drug or device that has not yet reached its primary endpoint.
31. Unable or unwilling to fulfill the protocol follow-up requirements.
32. Subject is a prisoner or member of other vulnerable populations.

Sub-study:

1. An inability to provide written informed consent for the sub-study.
2. Use of anti-hypertensive medications directly acting on the sympathetic nervous system, that cannot be discontinued safely.
3. Uncontrolled or involuntary movements disturbing microneurography, such as tremors, fasciculations, and chorea.
4. Absence or paralysis of both legs.
5. Polyneuropathy or clinical suspicion for autonomic nervous system dysfunction.
6. Known claustrophobia.
7. Metallic implants, prostheses or other foreign bodies causing potential artefacts obscuring the visibility of signals from the site of MobiusHD implantation during MRimaging.
8. Cochlear implants, pacemakers, neurostimulators, stents or grafts at risk of malfunction due to the magnetic field.

9. Underlying conditions that prohibit a Valsalva maneuver: i.e. aortic stenosis, cardiac arrhythmia, glaucoma, and/or retinopathy.

Day of procedure (angiography):

1. Evidence of any carotid plaque, ulceration or stenosis on selective carotid angiography performed in orthogonal views. Luminal diameters will be assessed to exclude subjects with ICA lumen diameters <5 mm or >11.75 mm within the planned location of the device placement.
2. Any angiographic evidence of plaque or ulceration in the aortic arch and/or the supra-aortic vasculature.
3. Inappropriate anatomy of the carotid bifurcation for deployment of the MobiusHD, including, but not limited to; tortuosity of the extracranial vessels and significant angulation of the common carotid artery bifurcation.
4. Type III arch or horizontal takeoff of the left carotid from the innominate and calcification of the carotid bulb.
