# Supplementary material for: Endovascular baroreflex amplification and the effect on sympathetic nerve activity in patients with resistant hypertension: A proof-of-principle study
Source: PLoS One. 2021 Nov 16;16(11):e0259826. doi: 10.1371/journal.pone.0259826 (PMC8594823; doi:10.1371/journal.pone.0259826)
Supplement: S2 File — (DOCX) [file pone.0259826.s003.docx]

**CALM-DIEM – CONTROLLING AND LOWERING BLOOD PRESSURE WITH THE MOBIUSHD™ – DEFINING EFFICACY MARKERS**

**Extensive Summary Study Protocol**

7 April 2016

**Table of contents**

[1. BACKGROUND 3](#_Toc85633205)

[2. METHODS 3](#_Toc85633206)

[2.1. Study design 3](#_Toc85633207)

[2.2. Study population 3](#_Toc85633208)

[2.2.1. Inclusion criteria 3](#_Toc85633209)

[2.2.2. Exclusion criteria 4](#_Toc85633210)

[2.3. Patient enrolment 5](#_Toc85633211)

[2.4. Intervention 6](#_Toc85633212)

[2.5. Study outcomes 8](#_Toc85633213)

[2.5.1. Primary study outcome 8](#_Toc85633214)

[2.5.2. Secondary study outcomes 8](#_Toc85633215)

[2.6. Study assessments 8](#_Toc85633216)

[2.6.1. Study visits 8](#_Toc85633217)

[2.6.1. Procedure 9](#_Toc85633218)

[2.1. Sample size 9](#_Toc85633219)

[2.2. Data analysis 11](#_Toc85633220)

[3. REFERENCES 11](#_Toc85633221)

# BACKGROUND

Worldwide prevalence estimates indicate that hypertension may affect as many as 1 billion individuals [1]. Hypertension is associated with an increased risk of myocardial infarction and stroke: for every 20 mmHg increase in systolic and every 10 mmHg increase in diastolic blood pressure (BP) the risk of cardiovascular disease doubles [2]. In the United States ~30% of adults are still unaware of their hypertension, ~41% are not on treatment and ~34% of those being treated are not controlled to BP levels <140/90 mmHg [1]. Therefore, treatment of hypertension should be optimized. For those patients with resistant hypertension (defined as elevated BP despite treatment with at least three antihypertensive drugs, including a diuretic) [3] with unsatisfactory response to antihypertensive medications, there is a need for new treatment strategies. New treatment strategies are aimed at reducing the activity of the sympathetic nervous system. Given the pivotal role of the carotid baroreflex in the autonomic nervous system, Vascular Dynamics’ approach is to modulate the baroreflex using a simple, passive, implantable device as a means to inhibit sympathetic signaling and consequently lower systemic BP. Based on data suggesting the potential utility of mechanical modulation of the baroreflex for lowering BP: canine studies [4, 5], acute BP changes in carotid artery stenting [6], and BP changes after variable neck pressure [7], Vascular Dynamics has developed the MobiusHD for the treatment of resistant hypertension. The MobiusHD, the subject of this protocol, is a sterile self-expanding nitinol implant, delivered through a disposable catheter, the delivery catheter, into the carotid artery. Together, the MobiusHD and delivery catheter make up the MobiusHD system.

# METHODS

## Study design

This is a prospective, multicenter study to be conducted at sites in countries where the MobiusHD system is commercially available. During the enrolment phase, up to 200 patients with primary resistant hypertension who have been selected for treatment with the MobiusHD, meet the protocol eligibility criteria, and consent to participation, will undergo placement of the MobiusHD implant in the carotid sinus and will be followed for 3 years.

The flow chart for patient screening and enrolment is provided in Figure 1. As shown, consented subjects are screened for determination of eligibility. All enrolled subjects who receive a MobiusHD implant will be examined at 7, 30, 90, 180, and 365 days, and 2 and 3 years post-implantation.

## Study population

### Inclusion criteria

Candidates for this study must meet the following criteria to be enrolled:

#### Screening

1. ≥18 years of age and ≤80 years of age.
2. Diagnosed with primary resistant hypertension.
3. Mean 24-hour systolic ambulatory BP ≥130 mmHg following at least 30 days on a stable antihypertensive medication regimen (no changes in medication or dose), and no more than 28 days prior to implantation.

### Exclusion criteria

Candidates will be ineligible for enrolment in the study if any of the following conditions are identified:

#### Screening

1. An inability provide written informed consent.
2. Known or clinically suspected baroreflex failure or autonomic neuropathy.
3. Known significant aortoiliac or common femoral artery disease that will prohibit safe femoral access.
4. Hypertension secondary to an identifiable and treatable cause other than sleep apnea (e.g. hyperaldosteronism, renal artery stenosis, pheochromocytoma, Cushing's disease, coarctation of the aorta, hyperparathyroidism and intracranial tumor).
5. Treatable cause of resistant hypertension including, but not limited to, improper BP measurement, volume overload and pseudotolerance (excessive sodium intake, volume retention from kidney disease, inadequate diuretic therapy), drug-induced or other causes (non-adherence, inadequate doses, inappropriate combinations, NSAIDs, COX-2 inhibitors, cocaine, amphetamines, other drugs, sympathomimetics, oral contraceptives, adrenocortical steroids, cyclosporine, tacrolimus, erythropoietin, excessive liquorice (including some chewing tobacco), ephedra, ma huang, bitter orange, and excessive alcohol intake).
6. Arm circumference >46 cm and/or BMI ≥45 kg/m2.
7. Chronic atrial fibrillation or recurrent atrial fibrillation with episode within the last 12 months.
8. History of bleeding complications with dual antiplatelet therapy in the past or known uncorrectable bleeding diathesis.
9. Current use of anticoagulation therapy, other than dual antiplatelet medications. Examples include vitamin K antagonists and direct-acting oral anticoagulants including apixaban, rivaroxaban, dabigatran and edoxaban.
10. Peptic ulcer disease with documented active ulcer or bleeding within the last year.
11. History of allergy to contrast media that cannot be managed medically.
12. Persistent symptomatic orthostatic hypotension (>20/10 mmHg).
13. Persistent symptomatic syncope documented to be related to hypertension within the last 6 months.
14. History of myocardial infarction or unstable angina within the past 3 months.
15. History of cerebral vascular accident (stroke or TIA) within the past year, and NIHSS >5 or mRS >1.
16. Chronic kidney disease (eGFR calculated by the Modification of Diet in Renal Disease equation <45 ml/min).
17. Prior carotid surgery, therapeutic radiation, or endovascular stent placement in either carotid region.
18. Severe valvular or structural heart disease (excluding left ventricular hypertrophy).
19. Severe chronic obstructive pulmonary disease (requiring 24-hour oxygen or oral steroids), asthma, or severe pulmonary hypertension.
20. Uncontrolled diabetes mellitus with HbA1c ≥10%.
21. Active infection within the last month requiring antibiotics.
22. Uncontrolled co-morbid medical condition, including mental health issues, that would adversely affect participation in the trial.
23. Co-morbid condition that reduces life expectancy to less than 1 year.
24. Planned surgery or other procedure within the next 6 months requiring cessation of antiplatelet medications.
25. Pregnant or lactating females. For females of child-bearing potential, a positive pregnancy test within 7 days of the screening visit or refusal to use a medically accepted method of birth control for the duration of the trial.
26. Carotid duplex studies demonstrating obstructive carotid disease, plaque, ulceration or >150 micron intima-media thickness (IMT) at the site of implantation and/or proximal to the carotid artery bulb and ≥50% disease distal to the carotid artery bulb, including the intracranial circulation.
27. Significant obstructive vascular disease, calcification or plaque of aortic arch and great vessels detected by ultrasound, computed tomography angiography (CTA) or magnetic resonance angiography (MRA).
28. Renal artery stenosis >50% or systolic gradient >10 mmHg in borderline cases diagnosed by renal artery imaging in the last 36 months. Acceptable renal artery imaging modalities include renal duplex, CTA, MRA, and selective or nonselective renal angiography depending on trial site diagnostic standards.
29. Internal carotid artery (ICA) lumen diameters <5 mm or >12.5 mm within the planned location of the implant placement via CTA or MRA. Evidence of landing zone restrictions, such as inadequate length, vessel tapering, and/or vessel curvature that would preclude safe placement of the implant.
30. Enrolled in a concurrent clinical trial of an investigational drug or device that has not yet reached its primary endpoint.
31. Unable or unwilling to fulfil the protocol follow-up requirements.
32. Subject is a prisoner or member of other vulnerable population.

#### Day of Procedure - Angiographic

1. Evidence of any carotid plaque, ulceration or any stenosis on selective carotid angiography performed in orthogonal views. Luminal diameters will be assessed to exclude subjects with ICA lumen diameters <5 mm or >11.75 mm within the planned location of the device placement.
2. Any angiographic evidence of plaque or ulceration in the aortic arch and/or the supra-aortic vasculature.
3. Inappropriate anatomy of the carotid bifurcation for deployment of the MobiusHD, including, but not limited to, tortuosity of the extracranial vessels and significant angulation of the common carotid artery bifurcation.
4. Type III arch or horizontal take-off of the left carotid from the innominate and any significant calcification of the carotid bulb.

## Patient enrolment

Recruitment may start after the clinical site obtained permission from the ethics committee and once the site initiation visit has been conducted. Patients with resistant hypertension will be recruited from Cardiology, Hypertension, or Nephrology clinics. The clinician will review the patient’s medical history for eligibility. Potential candidates will be fully informed of the purpose of the study and the nature of the implantation procedure. Once the patient’s potential eligibility has been determined, the investigator will discuss the study and ask the patient if he/she is interested in participating. Patients who voluntarily agree to participate will be asked to sign and date the written patient information form. The patient information form must be signed by the potential study participant before any patient data are recorded in the study database. All subjects who meet the eligibility criteria and give written informed consent are considered enrolled in the study.

Study participation is voluntary and patients may choose to withdraw consent for this study at any time, for any reason, without effect on subsequent medical treatment or relationship with treating physician. Additionally, the investigator may choose to withdraw patients from the study at any time if he feels that it is in the patient’s best interest to discontinue the study. There are no follow-up requirements for subjects who electively withdraw from the study prior to treatment with the MobiusHD implant. Patients with an ongoing adverse event (AE) at the time of withdrawal should be followed in the trial until the event has been resolved or stabilized. Withdrawn patients will be included in the outcome data analysis.

If the investigator is unable to place a MobiusHD implant in a subject, the subject will be followed to the 30-day visit and will then be exited from the study in the absence of any ongoing AEs. If an AE is ongoing or not stabilized, the subject will continue to be followed until complete resolution or stabilization of the AE with no expectation or need for further treatment. Patients receiving the MobiusHD implant will be followed up for 3 years.

## Intervention

The intervention investigated in this study is MobiusHD implantation. The MobiusHD is a self-expanding nitinol implant that is delivered intravascular and unilaterally to the carotid sinus via the delivery catheter. The MobiusHD implant is preloaded in the delivery catheter and collectively they make up the MobiusHD system. The MobiusHD implant is available in 3 sizes: A) vessel diameter 5.00-7.00 mm, B) 6.25-9.00 mm, and C) 8.00-11.75 mm.

All subjects that receive a MobiusHD implant will be required to be on dual antiplatelet therapy, as either:

- a daily dose of 81-100 mg aspirin and 75 mg clopidogrel for a minimum of 3 days prior to the day of procedure or;
- a 81-100 mg aspirin and 300 mg clopidogrel loading dose up to 2 hours after the procedure.

All subjects will be required to take a daily dose of 81-100 mg aspirin and 75 mg clopidogrel for 3 months post-procedure. Aspirin will continue for the duration of study participation. Substitutes for aspirin and clopidogrel can be used, but should be equivalent.


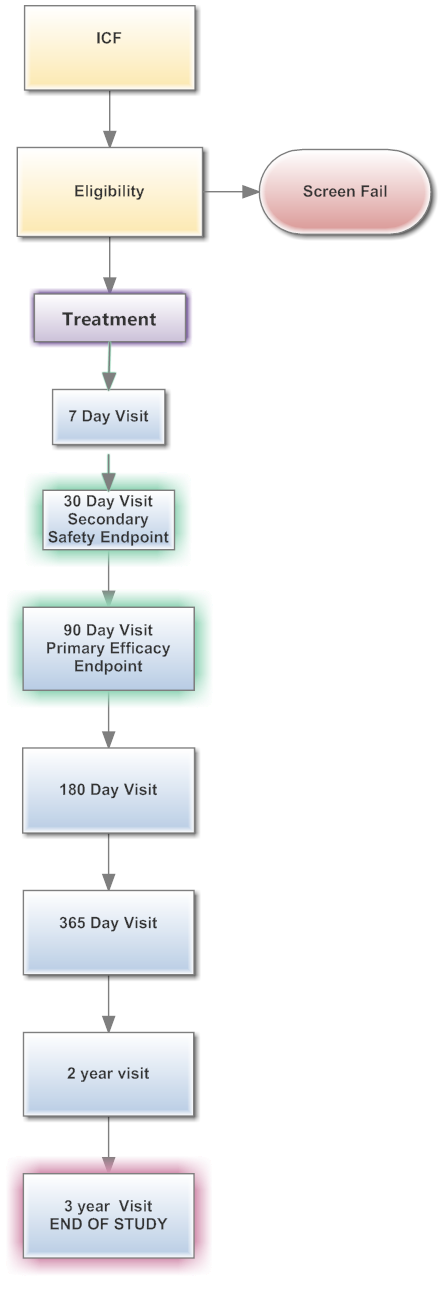


**Figure 1:** Flow chart showing the study assessments. ICF = informed consent form.

## Study outcomes

### Primary study outcome

Change in mean 24-hour ambulatory systolic BP from baseline to 90 days post implantation.

### Secondary study outcomes

#### Safety outcomes:

- 30-day major adverse clinical events (MACEs) including death, stroke, and/or myocardial infarction
- Peri-procedural device-related serious events (i.e. dissection, rupture, aneurysm)
- Serious adverse events (SAEs) and unanticipated adverse device effects (UADEs) during the first 3 years of follow-up

#### Efficacy outcomes:

- Change in mean 24-hour ambulatory systolic BP from baseline to 180 days and 365 days post implantation
- Change in heart rate
- Changes in antihypertensive medications/dosages
- Changes in Quality of Life (QoL) scores
- Changes in healthcare utilization

## Study assessments

### Study visits

The study assessments that are performed at baseline, pre-procedure, the day of procedure, the day after procedure, and during follow-up visits at 7, 30, 90, 180 and 365 days, and 1, 2 and 3 years post-procedure are summarized in Table 1.

#### Blood pressure measurement

BP measurements during study visits will be performed as follows:

- Office BP is measured with an oscillometric device, in sitting position, with an appropriately sized cuff, and on the arm that measures the highest BP. A minimum of two readings should be taken at intervals of at least one minute, and the average of those readings should be used to represent the patient's BP. If there is >5 mmHg difference between the first and second readings, additional one or two readings should be obtained, and then the average of these multiple readings is used.
- 24-hour ambulatory BP is measured with a validated oscillometric device on the non-dominant arm. At least 70% of the expected measurements over 24 hours should be recorded for the measurement to be valid.

#### Adverse events

An AE is defined as any untoward medical occurrence, unintended disease or injury or any untoward clinical sign (including an abnormal laboratory finding) in patients whether or not related to the medical device or procedure. An AE should be classified as serious (SAE) if it leads to death; a serious deterioration in the patient’s health; or fetal distress, fetal death, congenital abnormality or birth defect. An unanticipated adverse device effect (UADE) is defined as any SAE caused by or associated with the device, which was not previously identified in nature, severity or degree of incidence. All AEs will be characterized by the following criteria: event term, severity, expectedness, relationship to the device/procedure, outcome, and treatment or action taken. All SAEs and UADEs will be reviewed and adjudicated by the data safety monitoring board, which includes an interventional cardiologist, a neurologist, an interventional radiologist, two hypertension specialists, and a biostatistician.

### Procedure

During the procedure (MobiusHD implantation) the patient will be placed in the standard supine position on the imaging table and prepped and draped as for any standard carotid transcatheter diagnostic or therapeutic procedure. Intravenous heparin or its equivalent is given during the angiogram and implantation, according to local hospital protocols. The aortic arch is viewed during baseline angiography in the left anterior oblique projection. Selective carotid angiography of the target vessel for implant is performed in orthogonal views. If the aortic arch and carotid meet the inclusion criteria then the operator will proceed with the implant. A 6 Fr guide sheath or 8 Fr guiding catheter is advanced into the selected common carotid artery via the femoral artery over a 0.035 inch (0.9 mm) guidewire using standard carotid artery access techniques. The MobiusHD delivery system is advanced over a 0.014 inch (0.4 mm) guidewire into the carotid bulb where the device is deployed. The MobiusHD is implanted unilaterally on the anatomically best suited side with the device size selected based on carotid bulb diameters: 5.00-7.00 mm (A), 6.25-9.00 mm (B) and 8.00-11.75 mm (C). Post procedure angiography should be performed to evaluate proper implant positioning, apposition, and maintenance of the vessel lumen. Final angiograms should record the implanted device location and the dependent territory in at least two orthogonal planes.

Following the procedure, subjects will be monitored closely for significant change in status (i.e. bleeding, hematoma, peripheral pulsations, severe hypotension and severe hypertension). If BP drops below 90/50 mmHg with clinical symptoms or if the subject has symptoms with a relative BP drop from baseline then aggressive rescue should be provided to increase BP (i.e. intravenous fluids, vasopressors, withdrawal of anti-hypertensive medication).

If a new neurological deficit or a significant change in neurological status is determined using the NIHSS, the research team should perform a CT-scan or MRI to access for intracranial bleeding or ischemia.

## Sample size

The number of subjects to be enrolled in the study is up to 200. This sample size is estimated to be sufficient to yield data for analyses and to support future regulatory submissions. No formal sample size calculation is performed. It is anticipated that up to 50 centers will enrol in a timely fashion. Each study site will be expected to enrol at least 4 and up to 30 subjects.

**Table 1**: Schedule of study visits and assessments

| **Visit** | **Screening** | **Procedure** | **Discharge** | **7 Days (+2/-3 days)** | **30 Days (+/- 7 days)** | **90 Days (+/- 14 days)** | **180 Days (+/- 30 days)** | **365 Days (+/30 days)** | **2 Years (+/30 days)** | **3 Years (+/30 days)** | **Unscheduled Visits** |
| --- | --- | --- | --- | --- | --- | --- | --- | --- | --- | --- | --- |
| **Eligibility** | **✓** | **✓** |  |  |  |  |  |  |  |  |  |
| **Informed Consent** | **✓** |  |  |  |  |  |  |  |  |  |  |
| **Medical History** | **✓** |  |  |  |  |  |  |  |  |  |  |
| **Physical** | **✓** | **✓** |  | **✓** | **✓** | **✓** | **✓** | **✓** | **✓** | **✓** | **✓** |
| **Neurological Exam (NIHSS)** | **✓** | **✓** | **✓** | **✓** | **✓** | **✓** | **✓** | **✓** | **✓** | **✓** |  |
| **Concomitant medications** | **✓** | **✓** |  | **✓** | **✓** | **✓** | **✓** | **✓** | **✓** | **✓** | **✓** |
| **12 lead ECG** | **✓** |  |  |  |  | **✓** |  |  |  |  |  |
| **Blood Testing** | **✓** |  | **✓** |  |  |  |  |  |  |  |  |
| **Pregnancy Test†** | **✓** |  |  |  |  |  |  |  |  |  |  |
| **Patient Diary** | **✓** | **✓** |  | **✓** | **✓** | **✓** |  |  |  |  |  |
| **Duplex Carotid Imaging** | **✓** |  | **✓** | **✓** | **✓** |  |  | **✓^‡^** |  |  |  |
| **CT or MR Angiography** | **✓** |  |  |  |  |  |  |  |  |  |  |
| **Renal Artery CTA/MRA or Duplex** | **✓ǂ** | **✓*** |  |  |  |  |  |  |  |  |  |
| **24 hour ABPM** | **✓** |  |  |  |  | **✓** | **✓** | **✓** | **✓** | **✓** |  |
| **MobiusHD Placement** |  | **✓** |  |  |  |  |  |  |  |  |  |
| **Carotid Angiogram** |  | **✓** |  |  |  |  |  |  |  |  |  |
| **Office cuff BP Measurement** | **✓** | **✓** | **✓** | **✓** | **✓** | **✓** | **✓** | **✓** | **✓** | **✓** | **✓** |
| **Quality of Life Questionnaire** | **✓** |  |  | **✓** | **✓** | **✓** | **✓** | **✓** | **✓** | **✓** |  |
| **Healthcare Utilization** | **✓** |  |  | **✓** | **✓** | **✓** | **✓** | **✓** | **✓** | **✓** | **✓** |
| **Adverse Events** | **✓** | **✓** | **✓** | **✓** | **✓** | **✓** | **✓** | **✓** | **✓** | **✓** | **✓** |

**ǂ** If not performed in 36 months preceding screening.

*In patients without adequate pre-procedure work-up for renal artery stenosis, non-selective renal angiography should be performed on the day of procedure (day 0).

† Women of child-bearing age

‡ If patient exits study prematurely, assess carotid with duplex, when possible.

## Data analysis

This study is designed to evaluate the safety and performance of the MobiusHD system in subjects with resistant hypertension; no formal hypothesis testing is planned. Where appropriate, descriptive statistics will be used to tabulate and summarize outcomes. Continuous variables will be summarized as appropriate (mean ± standard deviation, median + range). Discrete variables will be summarized by frequencies and percentages. Adverse events will be summarized by presenting the number and percentage of patients having any adverse event. Any other information collected (such as severity or relationship to the device and/or procedure) will be listed as appropriate. Any statistical tests performed to explore the data will be used only to highlight any interesting comparisons that may warrant further consideration.

# REFERENCES

1. Chobanian AV, Bakris GL, Black HR, Cushman WC, Green LA, Izzo JL, Jr., et al. The Seventh Report of the Joint National Committee on Prevention, Detection, Evaluation, and Treatment of High Blood Pressure: the JNC 7 report. JAMA. 2003;289(19):2560-72.

2. Staessen JA, Gasowski J, Wang JG, Thijs L, Den Hond E, Boissel JP, et al. Risks of untreated and treated isolated systolic hypertension in the elderly: meta-analysis of outcome trials. Lancet. 2000;355(9207):865-72.

3. Calhoun DA, Jones D, Textor S, Goff DC, Murphy TP, Toto RD, et al. Resistant hypertension: diagnosis, evaluation, and treatment: a scientific statement from the American Heart Association Professional Education Committee of the Council for High Blood Pressure Research. Circulation. 2008;117(25):e510-26.

4. Bagshaw RJ, Barrer SJ. Effects of Angioplasty Upon Carotid-Sinus Mechanical-Properties and Blood-Pressure Control in the Dog. Neurosurgery. 1987;21(3):324-30.

5. Fadali. Enlarging the Lumen of the Carotid Sinus. an experimental Treatment of Systemic Arterial Hypertension. Archives of Surgery. 1969;99(5):624-7.

6. McKevitt FM, Sivaguru A, Venables GS, Cleveland TJ, Gaines PA, Beard JD, et al. Effect of treatment of carotid artery stenosis on blood pressure: a comparison of hemodynamic disturbances after carotid endarterectomy and endovascular treatment. Stroke. 2003;34(11):2576-81.

7. Fadel PJ, Ogoh S, Keller DM, Raven PB. Recent insights into carotid baroreflex function in humans using the variable pressure neck chamber. Exp Physiol. 2003;88(6):671-80.
