# Supplementary material for: Endovascular baroreflex amplification and the effect on sympathetic nerve activity in patients with resistant hypertension: A proof-of-principle study
Source: PLoS One. 2021 Nov 16;16(11):e0259826. doi: 10.1371/journal.pone.0259826 (PMC8594823; doi:10.1371/journal.pone.0259826)
Supplement: S3 File — (DOCX) [file pone.0259826.s004.docx]

**CALM-DIEM SUB-STUDY: STUDYING THE EFFECT OF THE MOBIUSHD ON SYMPATHETIC ACTIVITY AND BAROREFLEX SENSITIVITY**

**A Sub-Study to:**

**CALM-DIEM: CONTROLLING AND LOWERING BLOOD PRESSURE WITH THE MOBIUSHD™ - DEFINING EFFICACY MARKERS**

**Extensive Summary Sub-Study Protocol**

19 September 2016

**Table of contents**

[1. SUB-STUDY BACKGROUND 3](#_Toc85632699)

[1.1. Proposed mechanism of action of the MobiusHD device 3](#_Toc85632700)

[1.2. The baroreflex 3](#_Toc85632701)

[1.3. Sympathetic activity 3](#_Toc85632702)

[1.3.1. Muscle sympathetic nerve activity 3](#_Toc85632703)

[1.3.2. Functional magnetic resonance imaging 3](#_Toc85632704)

[1.4. Baroreflex sensitivity 4](#_Toc85632705)

[1.5. Enhancing sympathetic activity 5](#_Toc85632706)

[2. METHODS 6](#_Toc85632707)

[2.1. Sub-study design 6](#_Toc85632708)

[2.2. Sub-study population 7](#_Toc85632709)

[2.2.1. Sub-study inclusion criteria 7](#_Toc85632710)

[2.2.2. Sub-study exclusion criteria 8](#_Toc85632711)

[2.3. Patient enrolment 8](#_Toc85632712)

[2.4. Sub-study outcomes 8](#_Toc85632713)

[2.5. Sub-study measurements 9](#_Toc85632714)

[2.5.1. Muscle sympathetic nerve activity 9](#_Toc85632715)

[2.5.2. Cold pressor test 10](#_Toc85632716)

[2.5.3. Functional magnetic resonance imaging 10](#_Toc85632717)

[2.6. Sample size 11](#_Toc85632718)

[2.7. Data analysis 11](#_Toc85632719)

[3. REFERENCES 12](#_Toc85632720)

# SUB-STUDY BACKGROUND

## Proposed mechanism of action of the MobiusHD device

It is assumed that the MobiusHD device lowers blood pressure (BP) through activation of the carotid baroreflex and consequent inhibition of the sympathetic nervous system and decrease in peripheral vascular resistance. There is, however, no evidence available to date to support the fact that the MobiusHD device lowers BP via this mechanism. The assessments outlined in this sub-study are aimed to determine whether the proposed mechanism of action of the MobiusHD device is correct. Understanding the mechanism of action may offer clinicians more information to support decisions when selecting patients for treatment with the MobiusHD device.

## The baroreflex

The baroreflex consists of two arms: sympathetic and parasympathetic (or cardiac) nerve circuits. The two circuits share the same afferent, but have different efferent pathways. A rise in BP induces vascular stretch and increases baroreceptor activity which i) activates the parasympathetic nervous system decreasing heart rate, and ii) inhibits the sympathetic nervous system causing vasodilatation.

Baroreceptor afferents travel in the glossopharyngeal and vagus nerves and project to the caudal region of the nucleus tractus solitarius (NTS), which in turn stimulates neurons in the region of the caudal ventrolateral medulla (CVLM) [1]. This region inhibits excitatory neurons in the rostroventrolateral medulla (RVLM). The RVLM is critical for maintenance of vascular tone and arterial BP and is the primary output nucleus for muscle sympathetic nerve activity (MSNA).

## Sympathetic activity

### Muscle sympathetic nerve activity

MSNA is a direct measure of systemic sympathetic activity. MSNA is measured through microneurography, which is one of the most reliable methods to assess overall sympathetic drive [2-4]. All peripheral nerves contain sympathetic fibers which is why, theoretically, every peripheral nerve is suitable for measuring MSNA [5]. Because of its superficial location, the peroneal nerve is used most frequently.

Although MSNA is characterized by large inter-individual differences, several studies have shown that it has a strong intra-individual reproducibility over many years [3, 6] provided that the circumstances under which MSNA is measured remain the same. Therefore, MSNA is an appropriate measure to compare intra-individual differences before and after MobiusHD device implantation in the present study. In order to measure the exclusive effect of the MobiusHD implantation on MSNA, all other conditions affecting sympathetic activity should remain the same.

### Functional magnetic resonance imaging

During the last few years, investigators have gained interest in assessing the relationship between brain stem regions and sympathetic nerve activity in order to develop a better understanding of hypertension pathophysiology. Active cerebral regions can be identified using functional magnetic resonance imaging (fMRI). The standard technique used to generate images in fMRI is blood oxygenation level dependent (BOLD) imaging which is an indirect method that relies on regional differences in cerebral blood flow to delineate regional activity. Cerebral blood flow increases 2-6 seconds after brain tissues became more active, carrying oxygenated haemoglobin to the tissues. Oxygenated haemoglobin has different paramagnetic properties than deoxygenated haemoglobin and can therefore be visualized.

Since the advent of human fMRI in the early 1990’s, brain activation patterns during various challenges have been explored and areas involved in the sympathetic nervous system have been unravelled. Studies have identified multiple brain regions that respond to Valsalva maneuver and cold pressor challenges [7, 8]. Also, the BOLD response was found to be different in patients with obstructive sleep apnea (a disease in which sympathetic drive is increased) compared to controls [8].

Since MSNA represents central sympathetic drive, several investigators have related MSNA to cerebral activity on fMRI. Macefield et al. showed that increases in MSNA were associated with robust bilateral increases in signal intensity in areas corresponding to the RVLM [9]. Reciprocal decreases in signal intensity occurred in regions of the NTS and CVLM. Group analysis also revealed increases in signal intensity in the caudal pressor area, medullary raphe and dorsal motor nucleus of the vagus. The same research group investigated whether higher areas of the brain were also associated with MSNA [10]. Signal intensity and MSNA were positively correlated in multiple cortical areas, including regions of the salience network: the left mid-insula, bilateral dorsolateral prefrontal cortex, bilateral posterior cingulate cortex, bilateral precuneus, left dorsomedial hypothalamus and bilateral ventromedial hypothalamus. A functional connectivity map revealed coupling between activity in higher brain regions and premotor sympathetic neurons in the RVLM.

Since multiple brain regions involved in sympathetic activity have been identified by fMRI, the BOLD imaging technique is assumed to be a useful method to examine changes in these regions (localized in the brain stem and higher cerebral regions) in participants before and after MobiusHD implantation. The Valsalva maneuver will be used to stimulate the baroreceptors and enhance sympathetic activity during BOLD imaging.

## Baroreflex sensitivity

There are several techniques that have been used to measure baroreflex sensitivity. Baroreflex sensitivity can be measured at rest during spontaneous BP oscillations or after mechanical challenges (such as the Valsalva maneuver) causing large fluctuations in BP. The fundamental idea of these measurements is to assess the strength of the negative feedback loop relating baroreflex input (arterial BP) to output (MSNA or heart rate): the sympathetic and cardiac baroreflex sensitivity, respectively.

The Valsalva maneuver represents a natural challenge for the baroreceptors to decreased cardiac output (fall in BP) as a result of elevated intra-thoracic pressure. The Valsalva maneuver enhances intra-thoracic pressure by performing moderately forceful exhalation against a closed airway. The normal physiological response during the Valsalva maneuver consists of four phases (Figure 1) [11]:

1. Initial pressure rise during the first few seconds forces blood out of the pulmonary circulation to the left atrium, causing a mild increase in stroke volume.
2. Reduced venous return due to the elevated intra-thoracic pressure, causing a decrease in stroke volume and a fall in BP which is compensated by baroreflex efferents causing tachycardia, vasoconstriction and a rise in BP.
3. After pressure is released, the pulmonary vessels and aorta re-expand causing decreased filling of the left atrium and a slight decrease in stroke volume during a few seconds.
4. Normal venous return returns causing a rapid rise in cardiac output, often accompanied with an overshoot. Then, heart rate and BP return to normal.


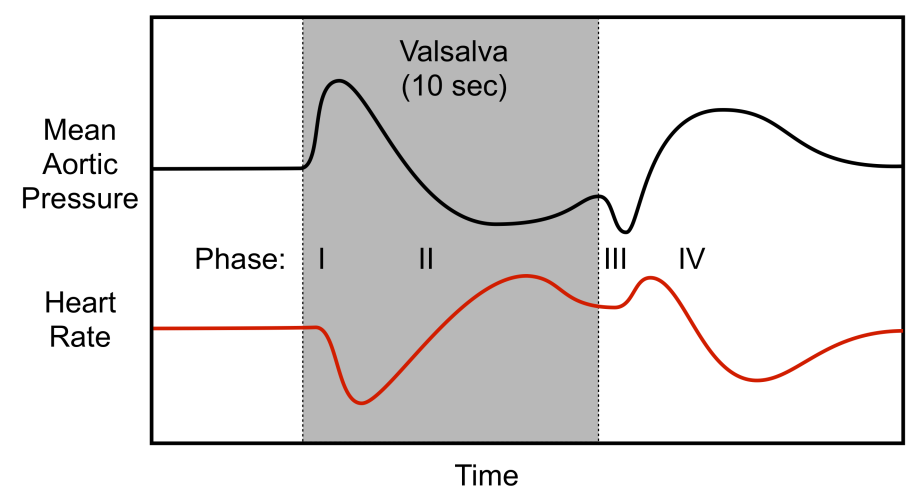


**Figure 1:** The four phases of the Valsalva maneuver. *Reproduced from* *Klabunde RE.* *Cardiovascular Physiology Concepts. Hemodynamics of a Valsalva maneuver. 2014. URL: https://www.cvphysiology.com/ Hemodynamics/H014.*

The relation between Valsalva maneuver and heart rate, BP and MSNA is visualized in Figure 2 [12]. Sympathetic baroreflex sensitivity is determined by relating MSNA bursts during the straining period of the Valsalva maneuver to the maximum fall of diastolic BP. Cardiac baroreflex sensitivity is determined by relating change in RR-interval to systolic BP changes during phase II (hypotensive stimulus) and phase IV (hypertensive stimulus) of the Valsalva maneuver.

## Enhancing sympathetic activity

There are several challenges that enhance sympathetic activity. One of these challenges is the Valsalva maneuver and is described above. Another challenge that activates the sympathetic nervous system is the cold pressor test (CPT).

In the 1980s Victor et al. provided direct evidence for increased sympathetic drive to skeletal muscles during a cold stimulus [13]. The CPT produces exaggerated sympathetic responses in hypertension-prone persons [14, 15]. Moreover, CPT responses are impaired in patients with orthostatic hypotension caused by efferent sympathetic failure [16]. Given that the MobiusHD is intended to alter sympathetic activity through modulation of the baroreflex, the present study also determines whether a patient’s response to a sympathetic stimulus (the CPT) changes after MobiusHD implantation.


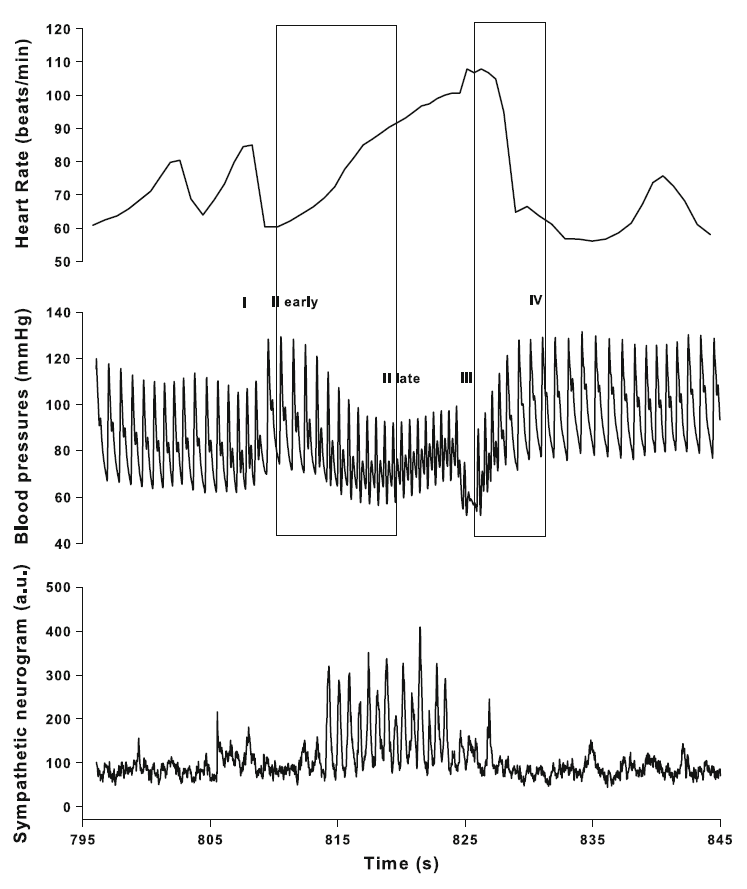


**Figure 2:** Heart rate, blood pressure and MSNA evolution during the Valsalva maneuver. *Reproduced from Yang H, Carter JR. Baroreflex sensitivity analysis: spontaneous methodology vs. Valsalva's maneuver. Clin Auton Res. 2013;23(3):133-9.*

# METHODS

## Sub-study design

The sub-study will be conducted at University Medical Center Utrecht as an integrated part of the CALM-DIEM study. A maximum of 16 subjects who consent to the CALM-DIEM clinical study will be additionally enrolled (after consent) in this sub-study. Only those subjects who pass screening CALM-DIEM study inclusion and exclusion criteria at CALM-DIEM study screening will undergo sub-study baseline assessments. Only those subjects who pass all CALM-DIEM study angiographic exclusion criteria on the CALM-DIEM study day of procedure will undergo the 90-day sub-study assessments.

The BP data collected within the CALM-DIEM study will be correlated with sympathetic activity and baroreflex sensitivity data collected at equivalent time points. A flow chart outlining how the sub-study integrates into the schedule of the CALM-DIEM study is shown in Figure 3. Subjects participating in the sub-study will undergo additional assessments at baseline and at 90-days post-implantation with the MobiusHD device.

**Figure 3:** Sub-study flow chart within the context of the main study schedule

## Sub-study population

Patients with primary resistant hypertension (24-hour mean ambulatory systolic BP >130 mmHg on a stable regimen of at least three antihypertensive medications, including a diuretic) who consented to participate in the regular CALM-DIEM study, are potential candidates for this sub-study.

### Sub-study inclusion criteria

Candidates must meet all of the following eligibility criteria to qualify for participation in the sub-study:

1. Eligible for the CALM-DIEM study and having passed all CALM-DIEM study inclusion and exclusion criteria at CALM-DIEM study screening.

### Sub-study exclusion criteria

Candidates will be ineligible for participation in the sub-study if any of the following conditions are identified during screening:

1. An inability to provide written informed consent for the sub-study.
2. Use of anti-hypertensive medications directly acting on the sympathetic nervous system, that cannot be discontinued safely.
3. Uncontrolled or involuntary movements disturbing microneurography, such as tremors, fasciculations and chorea.
4. Absence or paralysis of both legs.
5. Polyneuropathy or clinical suspicion for autonomic nervous system dysfunction.
6. Known claustrophobia.
7. Metallic implants, prostheses or other foreign bodies causing potential artefacts obscuring the visibility of signals from the site of MobiusHD implantation during MRI.
8. Cochlear implants, pacemakers, neurostimulators, stents or grafts at risk of malfunction due to the magnetic field.
9. Underlying conditions that prohibit a Valsalva maneuver: i.e. aortic stenosis, cardiac arrhythmia, glaucoma, and/or retinopathy.

## Patient enrolment

Recruitment may start after the clinical site obtained permission from the ethics committee and once the site initiation visit has been conducted. The clinician will review the patient’s medical history for eligibility. Potential candidates will be informed of the purpose of the sub-study and the nature of the additional assessments. Once the patient’s potential eligibility has been determined, the investigator will discuss the sub-study and ask the patient if he/she is interested in participating. Patients who voluntarily agree to participate will be asked to sign and date the written sub-study patient information form. The patient information form must be signed by the potential sub-study participant before any sub-study measurements are performed and before any patient data are recorded in the sub-study database.

Sub-study participation is voluntary, and patients may choose to withdraw consent for this sub-study at any time for any reason without effect on their participation in the study, subsequent medical treatment, or relationship with treating physician. Additionally, the investigator may choose to withdraw patients from the sub-study at any time if he feels that it is in the patient’s best interest to discontinue the sub-study. Withdrawn patients and sub-study subjects who fail to fulfil the CALM-DIEM study angiography eligibility criteria at the CALM-DIEM study day of procedure, may be replaced.

## Sub-study outcomes

1. Change in sympathetic activity from baseline to 90 days post-treatment measured by:
   1. MSNA (burst frequency and burst incidence)
   2. BOLD fMRI (resting state and task-based during the Valsalva maneuver)
2. Change in baroreflex sensitivity from baseline to 90 days post-treatment expressed as:
   1. Sympathetic baroreflex sensitivity: spontaneous and evoked (by the Valsalva maneuver)
   2. Cardiac baroreflex sensitivity: spontaneous and evoked (by the Valsalva maneuver)

## Sub-study measurements

### Muscle sympathetic nerve activity

The circumstances under which baseline and 3-month MSNA are measured, will be kept the same regarding factors that influence sympathetic activity. Antihypertensive medications that directly influence sympathetic activity will be discontinued for 2 weeks. This is because a stable medication regime cannot be guaranteed as it can be necessary to reduce antihypertensive medications after MobiusHD implantation. We believe it is safe to stop these medications in this population since these patients have already shown to be able to stop their prescribed regimen without developing any problems during the regular diagnostic work-up in our center.

Dr. P. Liam Oey, who is experienced in conducting microneurography for investigational purposes, will be the neuro-electrophysiologist in this project to perform the procedure. During the time of the procedure the subject will be in supine position. Intraneural recording is made with a tungsten microelectrode with a shaft of 0.2 mm and a tip of a few micrometers placed in the peroneal nerve, at the fibular head, just below the knee (Figure 4) [5]. A reference electrode will be placed subcutaneously within 2-3 cm of the former. The correct position of the electrode will be confirmed by the characteristic response to a Valsalva maneuver (increasing MSNA, shown in Figure 2) and by exposing the patient to sudden noises (creating a fright reaction, not supposed to affect MSNA).

After the MSNA signal has been identified, a 10 minute period of rest will be required in order to stabilize the reading. Subsequently, MSNA will be recorded during a 5-minute resting measurement followed by 3 times three Valsalva challenges, the CPT (consisting of a 2-minute pre-CPT, 2-minute actual CPT and 3-minute post-CPT measurement), and finally a second 5-minute resting measurement. If a Valsalva maneuver fails, the subject will be asked to repeat the block of three Valsalva challenges. During the MSNA measurements continuous BP as well as respiration and heart rate recordings will be captured using a non-invasive calibrated device.

**Figure 4:** placement of the active electrode and reference electrode at the peroneal nerve. *Reproduced from* *White DW, Shoemaker JK, Raven PB. Methods and considerations for the analysis and standardization of assessing muscle sympathetic nerve activity in humans. Auton Neurosci. 2015;193:12-21.*


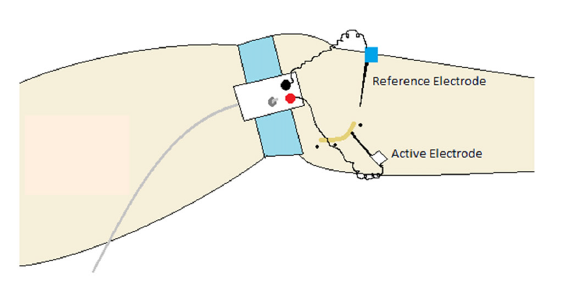


### Cold pressor test

During the CPT, the subject will be instructed to immerse his hand (up to the wrist) in ice water for 2 minutes. Recordings will be done up to 3 minutes after the CPT, as it has been shown that MSNA activity (and therefore also sympathetic activity) normalizes after this period, see Figure 5 [13, 17]. Patients will be told to avoid either (i) isometric contraction, (ii) performing a Valsalva maneuver, or (iii) holding expiration during the CPT.

**Figure 5:** MSNA response to CPT stimulus. The left column shows the response in patients before and after treatment with hydrochlorothiazide. The right column shows the response in patients before and after treatment with aliskiren. The top row depicts MSNA total activity and the bottom row MSNA burst frequency. BL = baseline, CPT = cold pressor test (2 minutes), REC = recovery time (3 minutes) *Reproduced from Jarvis SS, Okada Y, Levine BD, Fu Q. Central integration and neural control of blood pressure during the cold pressor test: a comparison between hydrochlorothiazide and aliskiren. Physiological Reports. 2015;3(9).*


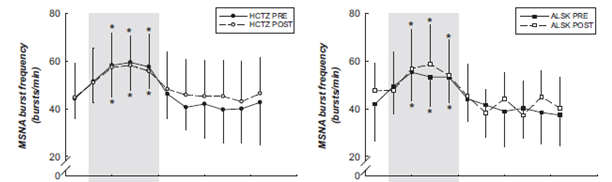

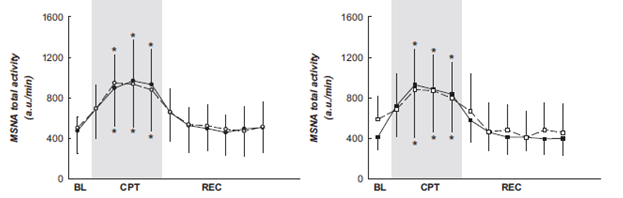


### Functional magnetic resonance imaging

Brain fMRI will be conducted by using the 3 Tesla MRI scan. A multiple echo scan sensitive to BOLD contrast, encompassing the entire brainstem and cerebral and cerebellar cortex, will be used. Before the subjects enter the fMRI scanning room, they will be carefully instructed how to perform the Valsalva maneuver, in order to minimize motion. Patients will lay supine and will receive a headphone (for communication with the control room) and ear plugs (to reduce noise). In order to improve signal to noise ratio, the patient’s head will be stabilized by foam pads. A pulse oximeter, 4-lead ECG, and breath registration device will be applied.

First a scout scan and an anatomic scan will be obtained, in order to relate BOLD signal changes to specific brain structures. Subsequently a perfusion scan for quantifying cerebral blood flow will be obtained. Thereafter, a resting state fMRI will be obtained, followed by task-based fMRI recordings: three blocks of three Valsalva maneuvers (15 seconds of strain followed by a period of rest in order to stabilize the body). The applied strain will be varied randomly among the blocks: one block in which a pressure of <5 mmHg is applied (as a control setting), one block of 20 mmHg and one block of 30 mmHg. The Valsalva maneuver will be performed by using a mouthpiece and bacterial filter fixed to ventilation tubing and connected to an analogue sphygmomanometer to measure the applied pressure. The mouthpiece, bacterial filter and plastic tubing are MRI compatible. The analogue sphygmomanometer will be deviated outside the MRI scan to the control room. Patients will be instructed through audio-visual feedback to inform whether they are bearing down too forcefully or not.

The MobiusHD device is determined to be MR conditional. The parameters used during fMRI have been determined to be within the bounds of the conditions set out in the MobiusHD information for use.

## Sample size

The sample size calculation is based on the primary endpoint, MSNA. The study is not powered for changes in BOLD fMRI. A limitation of the sample size calculation is that the effect of the MobiusHD device on MSNA is currently unknown. The change in MSNA is anticipated to be higher than the moxonidine effect in the population investigated by Dorresteijn et al. (burst frequency -5.9 ± 3.1 bursts/min and burst incidence -7.0 ± 4.6 bursts/100 beats) [18] and comparable with the barostimulator effect in the population investigated by Gronda et al. (burst frequency -12.5 ± 4.3 bursts/min and burst incidence -19.5 ± 9.3 bursts/100 beats) [19]. Therefore, an assumed change of 10 bursts/min and 15 bursts/100 beats seems realistic. To obtain 90% power of detecting a change of 10 burst/min (with assumed SD of 5) and 15 bursts/100 beats (with assumed SD of 9) with 95% confidence (2-sided), 9 pairs of measurements will be needed (powered on burst incidence).

Based on data from prior MSNA studies in our hospital, we know that ~15% of the microneurography measurements will fail (not being able to find a signal) [18, 20-22] and that 15% of the patients do not want to undergo a second microneurography [18, 21]. To account for this, 16 inclusions will be required. Calculation: 9 / 0.85 (15% of the measures fail) / 0.70 (15% of the measures fail and 15% of the patients do not want to undergo a second measurement) = 16. If a set of 9 MSNA measurements has been obtained, no more patients will be included and the amount of 16 inclusions does not have to be met. All implanted subjects in which a successful MSNA measurement has been obtained at baseline, will have a second MSNA measurement at 90 days. Moreover, all implanted patients who were scanned at baseline will have a second fMRI after 90 days.

## Data analysis

Descriptive data will be presented as categorical (n, %), normally distributed continuous (mean ± standard deviation), or non-normally distributed continuous variables (median, interquartile range), as appropriate.

Change in spontaneous MSNA will be reported as the mean change in burst frequency (bursts/minute) and burst incidence (bursts/100 heartbeats). Evoked sympathetic baroreflex sensitivity will be expressed as change in MSNA / change in diastolic BP (during phase II of the Valsalva maneuver). Evoked cardiac baroreflex sensitivity will be expressed as change in RR-interval / change in systolic BP (during phase II and phase IV of the Valsalva maneuver). For quantification of sympathetic and cardiac baroreflex sensitivity during spontaneous BP changes, linear regression will be performed. Change in MSNA, MAP and HR response to CPT will be reported as the mean change in i) MSNA (burst frequency and burst incidence), ii) MAP and iii) HR enhancement during the CPT compared to before CPT. Change in brain stem and cerebral BOLD signal intensity will be evaluated for every region of interest for spontaneous and evoked activity.

Mean changes will be evaluated by the Paired Samples T-Test (if normally distributed) or the nonparametric Wilcoxon Signed-Ranks Test (if not normally distributed) with corresponding 95% confidence intervals. Unless otherwise specified a two-sided 0.05 level of significance will be used to show a significant change.

# REFERENCES

1. Wehrwein EA, Joyner MJ. Regulation of blood pressure by the arterial baroreflex and autonomic nervous system. Handb Clin Neurol. 2013;117:89-102.

2. Sundlof G, Wallin BG. The variability of muscle nerve sympathetic activity in resting recumbent man. J Physiol. 1977;272(2):383-97.

3. Fagius J, Wallin BG. Long-term variability and reproducibility of resting human muscle nerve sympathetic activity at rest, as reassessed after a decade. Clin Auton Res. 1993;3(3):201-5.

4. Grassi G, Mark A, Esler M. The sympathetic nervous system alterations in human hypertension. Circ Res. 2015;116(6):976-90.

5. White DW, Shoemaker JK, Raven PB. Methods and considerations for the analysis and standardization of assessing muscle sympathetic nerve activity in humans. Auton Neurosci. 2015;193:12-21.

6. Kimmerly DS, O'Leary DD, Shoemaker JK. Test-retest repeatability of muscle sympathetic nerve activity: influence of data analysis and head-up tilt. Auton Neurosci. 2004;114(1-2):61-71.

7. Henderson LA, Macey PM, Macey KE, Frysinger RC, Woo MA, Harper RK, et al. Brain responses associated with the Valsalva maneuver revealed by functional magnetic resonance imaging. J Neurophysiol. 2002;88(6):3477-86.

8. Harper RM, Macey PM, Henderson LA, Woo MA, Macey KE, Frysinger RC, et al. fMRI responses to cold pressor challenges in control and obstructive sleep apnea subjects. J Appl Physiol (1985). 2003;94(4):1583-95.

9. Macefield VG, Henderson LA. Real-time imaging of the medullary circuitry involved in the generation of spontaneous muscle sympathetic nerve activity in awake subjects. Hum Brain Mapp. 2010;31(4):539-49.

10. James C, Macefield VG, Henderson LA. Real-time imaging of cortical and subcortical control of muscle sympathetic nerve activity in awake human subjects. Neuroimage. 2013;70:59-65.

11. R.E. K. Hemodynamics of a Valsalva maneuver. 2014 [updated 4-28-2014. Available from: <https://www.cvphysiology.com/Hemodynamics/H014>.

12. Yang H, Carter JR. Baroreflex sensitivity analysis: spontaneous methodology vs. Valsalva's maneuver. Clin Auton Res. 2013;23(3):133-9.

13. Victor RG, Leimbach WN, Jr., Seals DR, Wallin BG, Mark AL. Effects of the cold pressor test on muscle sympathetic nerve activity in humans. Hypertension. 1987;9(5):429-36.

14. Briggs FJO, H. Vasomotor response of normal and hypertensive individuals to thermal stimulus (cold). Minn Med. 1933;16:481-6.

15. Hines EA, Brown GE. The cold pressor test for measuring the reactibility of the blood pressure data concerning 571 normal and hypertensive subjects. Am Heart J. 1936;11:1-9.

16. Disorders of the autonomic nervous system [press release]. Oxford: Blackwell Press1974.

17. Jarvis SS, Okada Y, Levine BD, Fu Q. Central integration and neural control of blood pressure during the cold pressor test: a comparison between hydrochlorothiazide and aliskiren. Physiological Reports. 2015;3(9).

18. Dorresteijn JA, Schrover IM, Visseren FL, Scheffer PG, Oey PL, Danser AH, et al. Differential effects of renin-angiotensin-aldosterone system inhibition, sympathoinhibition and diuretic therapy on endothelial function and blood pressure in obesity-related hypertension: a double-blind, placebo-controlled cross-over trial. J Hypertens. 2013;31(2):393-403.

19. Gronda E, Seravalle G, Brambilla G, Costantino G, Casini A, Alsheraei A, et al. Chronic baroreflex activation effects on sympathetic nerve traffic, baroreflex function, and cardiac haemodynamics in heart failure: a proof-of-concept study. Eur J Heart Fail. 2014;16(9):977-83.

20. Klein IHHT, Ligtenberg G, Oey PL, Koomans HA, Blankestijn PJ. Enalapril and losartan reduce sympathetic hyperactivity in patients with chronic renal failure. J Am Soc Nephrol. 2003;14(2):425-30.

21. Siddiqi L, Oey PL, Blankestijn PJ. Aliskiren reduces sympathetic nerve activity and blood pressure in chronic kidney disease patients. Nephrol Dial Transpl. 2011;26(9):2930-U1514.

22. Siddiqi L, Joles JA, Oey PL, Blankestijn PJ. Atorvastatin reduces sympathetic activity in patients with chronic kidney disease. Journal of Hypertension. 2011;29(11):2176-80.
