# Supplementary figures and images for: Endovascular baroreflex amplification and the effect on sympathetic nerve activity in patients with resistant hypertension: A proof-of-principle study
Source: PLoS One. 2021 Nov 16;16(11):e0259826. doi: 10.1371/journal.pone.0259826 (PMC8594823; doi:10.1371/journal.pone.0259826)

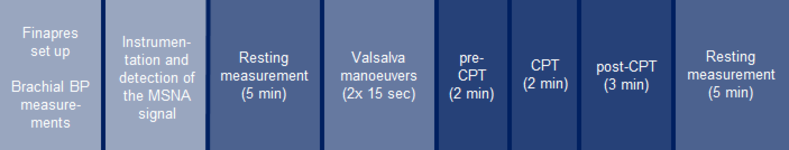

Supplement: S1 Fig — BP = blood pressure, MSNA = muscle sympathetic nerve activity, CPT = cold pressor test. (TIF) [file pone.0259826.s006.tif]

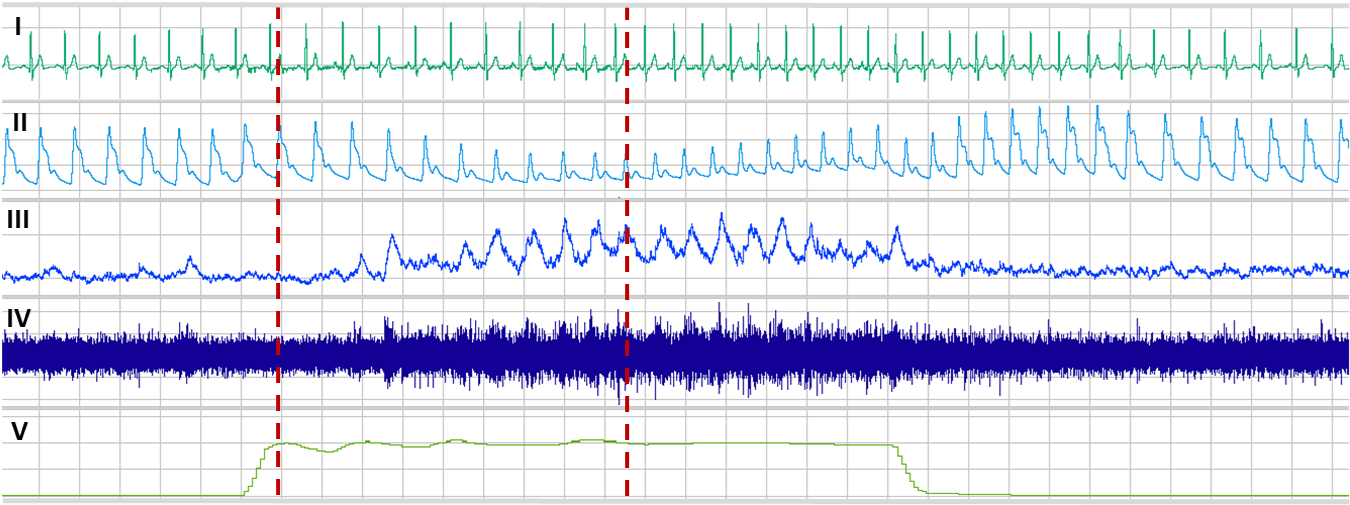

Supplement: S2 Fig — Heart rate (I), beat-to-beat blood pressure (II), integrated MSNA (III), filtered MSNA (IV), and pressure curve of the mouth pressure applied during the Valsalva manoeuver (V). The part between the vertical dashed lines represents early phase II of the Valsalva manoeuver. (TIF) [file pone.0259826.s007.tif]
