# Supplementary material for: Endovascular baroreflex amplification and the effect on sympathetic nerve activity in patients with resistant hypertension: A proof-of-principle study
Source: PLoS One. 2021 Nov 16;16(11):e0259826. doi: 10.1371/journal.pone.0259826 (PMC8594823; doi:10.1371/journal.pone.0259826)
Supplement: S1 Table — N represents the number of patients excluded from the 13 patients who underwent 3-month follow-up measurements. BRS = baroreflex sensitivity, cBRS = cardiac BRS, xBRS = cross-correlation BRS, sBRS = sympathetic BRS, T50 = diastolic BP at which 50% of the heartbeats is associated with a burst, CPT = cold pressor test, MAP = mean arterial pressure, HR = heart rate, NA = not applicable, BP = blood pressure, SBP = systolic BP, DBP = diastolic BP, HRV = heart rate variability, LF = low frequency, HF = high frequency. (PDF) [file pone.0259826.s008.pdf]

	N	reasons for exclusion
**MSNA**		
Burst frequency (bursts/min)<br>Burst incidence (bursts/100hb)<br>Mean beat-to-beat median spike frequency (spikes/sec)<br>Mean beat-to-beat spike count (spikes/beat)	3	insufficient quality (2)<br>ventricular extrasystoles (1)
**BRS**		
cBRS Valsalva (msec/mmHg)	1	ventricular extrasystoles (1)
cBRS Sequence up (msec/mmHg)<br>cBRS Sequence down (msec/mmHg)<br>xBRS (msec/mmHg)	3	ventricular extrasystoles (1)<br>arrhythmia (1)<br>not ≥3 significant sequences (1)
sBRS Valsalva (spikes/sec/mmHg)	5	insufficient quality (4)<br>ventricular extrasystoles (1)
sBRS T_{50} (mmHg)<br>sBRS treshold slope (%/mmHg)	4	insufficient quality (2)<br>slope not significant (1)<br>ventricular extrasystoles (1)
**CPT responses**		
Δ MAP to CPT (mmHg)<br>Δ HR to CPT (bpm)	0	NA
Δ burst incididence to CPT (bursts/100hb)<br>Δ burst frequency to CPT (bursts/min)	5	insufficient quality (4)<br>ventricular extrasystoles (1)
**BP and HR**		
Office SBP (mmHg)<br>Office DBP (mmHg)<br>24-h Ambulatory SBP (mmHg)<br>24-h Ambulatory DBP (mmHg)<br>HR (bpm)	0	NA
**HRV**		
Total power ($msec^2$)<br>LF Power ($msec^2$)<br>HF Power ($msec^2$)	2	ventricular extrasystoles (1)<br>arythmia (1)

N represents the number of patients excluded from the 13 patients who underwent 3-month follow-up measurements. BRS = baroreflex sensitivity, cBRS = cardiac BRS, xBRS = cross-correlation BRS, sBRS = sympathetic BRS, T_{50} = diastolic BP at which 50% of the heartbeats is associated with a burst, CPT = cold pressor test, MAP = mean arterial pressure, HR = heart rate, NA = not applicable, BP = blood pressure, SBP = systolic BP, DBP = diastolic BP, HRV = heart rate variability, LF = low frequency, HF = high frequency.
