# Supplementary material for: Endovascular baroreflex amplification and the effect on sympathetic nerve activity in patients with resistant hypertension: A proof-of-principle study
Source: PLoS One. 2021 Nov 16;16(11):e0259826. doi: 10.1371/journal.pone.0259826 (PMC8594823; doi:10.1371/journal.pone.0259826)
Supplement: S2 Table — DM 2 = diabetes mellitus type 2, CVD = history of cardiovascular disease, RDN = history of renal denervation, mn b2b = mean beat-to-beat, f = female, m = male, A = ACE-inhibitor or angiotensin-II-receptor blocker, C = calcium antagonist, D = diuretic, MRA = mineralocorticoid receptor antagonist, α = alpha blocker, β = beta blocker, R-i = direct renin inhibitor. (PDF) [file pone.0259826.s009.pdf]

patient number	age	sex	BMI	eGFR	DM	CVD	RDN	medication baseline	escape medication	burst frequency (bursts/min)	burst incidence (bursts/100hb)	mn b2b median spike freq (spikes/sec)	mn b2b spike count (spikes/beat)	office SBP (mmHg)	office DBP (mmHg)	Δ medication	Δ burst frequency (bursts/min)	Δ burst incidence (bursts/100hb)	Δ mn b2b median spike freq (spikes/sec)	Δ mn b2b spike count (spikes/beat)	Δ office SBP (mmHg)	Δ office DBP (mmHg)
1	55	m	31	90	+	-	+	A, C, D	-	36.7	41.7	8.3	8.7	212	115	-	8.6	5.5	0.5	1.4	-51	-23
2	48	m	25	90	-	-	-	A, C, D, MRA	-	15.7	21.2	6.5	9.1	203	128	-	-9.3	-11.9	-1.5	-1.0	-33	-15
3	41	m	34	90	-	-	+	A, C, D	-	12.2	18.7	5.5	6.1	173	98	-	·	·	·	·	-7	6
4	48	f	20	79	-	-	-	A, C, α	amlodipine 10mg	17.8	28.3	15.3	11.6	184	115	-α	·	·	·	·	-30	-15
5	51	m	27	61	-	-	-	A, C, D, MRA	amlodipine 5mg	31.1	39.8	14.0	14.2	189	131	-	-11.1	-2.3	-10.0	-9.4	-15	-11
6	49	f	27	90	+	-	-	A, C, D, β, α	amlodipine 10mg	·	·	·	·	194	117	-½C, -D, -β, -α	·	·	·	·	·	·
7	56	f	25	90	-	-	-	A, C, D, α	diltiazem 200mg	·	·	·	·	189	112	-½C, -D, -α	·	·	·	·	-39	-19
8	63	m	26	79	-	-	-	A, C, D	-	38.9	57.8	8.1	9.7	150	78	-	-23.6	-35.3	-1.0	-2.2	9	9
9	44	m	36	55	-	-	-	A, C, MRA, α, R-i	amlodipine 10mg	70.6	81.2	18.5	18.4	210	136	-	-4.6	-6.0	-5.5	-7.1	-5	0
10	65	f	20	90	-	-	+	A, C, D, α	amlodipine 10mg	47.4	55.7	16.8	14.8	188	125	-	-6.4	-2.7	-3.7	-3.2	-30	-32
11	50	m	30	90	-	-	-	C, D, R-i	-	33.0	36.6	13.1	11.3	154	97	-	0.2	4.5	-4.3	-1.5	-1	3
12	56	m	31	75	-	+	+	A, C, D	amlodipine 10mg	44.4	78.6	18.2	20.4	187	105	-	-13.6	-21.3	-8.8	-7.4	19	0
13	51	m	26	65	-	-	-	A, C, D	amlodipine 5mg	18.2	28.3	12.6	16.3	192	123	-	15.3	16.8	-6.1	-4.3	-1	21
14	46	m	31	90	-	-	-	A, D, MRA	amlodipine 5mg	47.4	71.6	15.9	21.8	142	90	-	3.5	15.0	12.6	5.1	-4	-4

DM 2 = diabetes mellitus type 2, CVD = history of cardiovascular disease, RDN = history of renal denervation, mn b2b = mean beat-to-beat, f = female, m = male, A = ACE-inhibitor or angiotensin-II-receptor blocker, C = calcium antagonist, D = diuretic, MRA = mineralocorticoid receptor antagonist, α = alpha blocker, β = beta blocker, R-i = direct renin inhibitor.
