# Supplementary material for: Endovascular baroreflex amplification and the effect on sympathetic nerve activity in patients with resistant hypertension: A proof-of-principle study
Source: PLoS One. 2021 Nov 16;16(11):e0259826. doi: 10.1371/journal.pone.0259826 (PMC8594823; doi:10.1371/journal.pone.0259826)
Supplement: S3 Table — *Only those related or possibly related to the device or procedure or with relatedness unknown. ˟Decrease in eGFR of >10%. (PDF) [file pone.0259826.s010.pdf]

	Number of patients	% of implanted patients
30-day major adverse clinical events		
Death	0	0%
Stroke	1	7%
Myocardial infarction	0	0%
Periprocedural device-related serious events	0	0%
Unanticipated adverse device effects	0	0%
Serious adverse events		
Groin bleeding requiring prolonged hospitalization	1	7%
Non-serious adverse events*		
Groin bleeding	1	7%
Temporary decrease in kidney function[×]	3	21%
Pain at puncture site	2	14%
Dizziness	1	7%
Epistaxis and axilary hematoma	1	7%
Headache	1	7%
Periprocedural chest pain	1	7%

*only those related or possibly related to the device or procedure or with relatedness unknown

[×]decrease in eGFR of >10%
